# Habbe Gule Aakh prevents glycolytic program and alleviates disease progression in a rheumatoid arthritis animal model

**DOI:** 10.3389/fimmu.2025.1633061

**Published:** 2025-08-29

**Authors:** Arulkumaran Rithvik, Shangomitra Bhattacharjee, Manshi Kumari Gupta, C. Sudandiradoss, Abdul Wadud, Mahaboobkhan Rasool

**Affiliations:** ^1^ Immunopathology Lab, School of Biosciences and Technology, Vellore Institute of Technology (VIT), Vellore, Tamil Nadu, India; ^2^ Department of Biotechnology, School of Biosciences and Technology, Vellore Institute of Technology (VIT), Vellore, Tamil Nadu, India; ^3^ Regional Research Institute of Unani Medicine, Srinagar, Central Council for Research in Unani Medicine (CCRUM), Ministry of AYUSH, Srinagar, India

**Keywords:** rheumatoid arthritis, fibroblast-like synoviocytes, Habbe Gule Aakh, toll-like receptor 4, glycolysis, TIMP1

## Abstract

**Background:**

The acquired tumorigenic phenotype of the resident fibroblast like synoviocytes (FLS) is cornerstone to exacerbating rheumatoid arthritis (RA) disease progression. Toll like receptor 4 (TLR4) signalling can sustain the proliferative and invasive phenotype of these synoviocytes resulting in cartilage degradation and bone damage. A marked increase in glycolytic activity also contributes to the malignant character of these cells. Herein, we aim to study the prospects of TLR4 activation leading to improved glycolytic flux. Further, we also strategize the therapeutic modality of Habbe Gule Aakh (HGA), a polyherbal unani formulation to rescue disease progression via blockade of TLR4 activation.

**Methods:**

We activated TLR4 signaling in SW982 cells, cultured in high glucose medium. Initially, the expression profile of glycolytic rate limiting enzymes- hexokinase 2 (HK2) and pyruvate kinase M2 (PKM2) was assessed. Next, we evaluated the ability of HGA to regulate the expression of these enzymes via ablation of TLR4 activation. Further, we investigated the pathway of glucose uptake via tissue inhibitor of matrix metalloproteinase 1 or TIMP1 and counterintuitively investigated HGA to arrest the uptake of glucose via p65-TIMP1 signaling axis. To sufficiently validate our findings, we utilised network pharmacology approach, to uncover the interactome of HGA against rheumatoid arthritis targets. Ultimately, we leveraged in-vivo models to support the anti-arthritic claims of HGA.

**Results:**

HGA regulated the proliferation and invasive phenotype of SW982 cells cultured in high glucose medium via blockade of TLR4 activation. Further, in-silico and in-vivo approaches suggest a mechanistic insight to the anti-arthritic activity of HGA upon blockade of TLR4-mediated glycolytic flux in resident synoviocytes.

**Conclusion:**

Pharmacological intervention with Habbe Gule Aakh can rescue exacerbation of rheumatoid arthritis disease severity via TLR4 signaling axis. The findings of this study strengthen the rationale for the use of HGA in clinical settings involving RA patients.

## 1 Introduction

Rheumatoid arthritis is a debilitating autoimmune disorder affecting the joints, with a global prevalence of 0.5 1% (1). Clinical manifestations of rheumatoid arthritis include symmetrical joint pain and inflammation (2). Fibroblast-like synoviocytes, which are the resident cells of the RA synovium, cause major damage to knee joints via their altered tumorigenic phenotype (3). Leveraging therapeutic strategies to combat synovial hyperplasia and cartilage degradation by targeting resident cells is a popular approach for combating disease severity (4). Recent studies have underscored the role of altered metabolic profiles of RA fibroblast-like synoviocytes (RA-FLS) in their acquired tumorigenic or pro-inflammatory characteristics. For instance, aberrant glycolytic programs are key factors that drive the pathogenicity of RA-FLS (5, 6). Thus, regulating the improved glycolytic program of resident synoviocytes in RA might prove to be an effective strategy to diminish disease progression.

Metabolic profiling of cells in the synovium of RA patients indicates a heightened glycolytic program, resulting in a pro-inflammatory phenotype (5). Recent reports have also indicated an improved glycolytic program in cells that exhibit improved plasticity or turnover rates. Single-cell RNA sequencing of RA-FLS suggests an escalated glycolytic program, with many cells expressing higher levels of glycolytic pathway enzymes, including hexokinase 2 (HK2), glucose transporter 1 (GLUT1), tissue inhibitor of matrix metalloproteinase 1 (TIMP1), and hypoxia-inducible factor 1 alpha (HIF1α) (7). These findings inspired us to investigate the altered glycolytic program in human RA synoviocytes as a popular approach to mitigate disease severity.

Toll-like receptor 4 (TLR4), which is expressed in chondrocytes, osteoblasts, and synoviocytes, assumes greater significance in the pathophysiology of rheumatoid arthritis. Accordingly, the TLR4 signaling pathway has been implicated in the induction of a pro-inflammatory phenotype in RA. Recent reports have suggested targeting TLR4 activation as an effective modality for RA management (8, 9). Previous reports from our laboratory have validated improved preclinical outcomes by targeting TLR4 via TAK-242 (resatorvid), a cell-permeable TLR4 antagonist (10). Similarly, TLR4 antagonists have been approved for the management of various pathological outcomes, including sepsis and lung inflammation (11, 12). Importantly, TLR4 has been implicated as an important upstream mediator of pathological events in RA, including M1 macrophage polarization and osteoclastogenesis (13, 14). Thus, investigating TLR4 as a key upstream trigger or initiator of the glycolytic program in synovial cells can offer new insights into RA disease management. Previous studies have suggested TLR4 pathway activation to improved glycolytic turnover. For instance, activation of TLR4 signaling pathway in dendritic cells and macrophages have promoted metabolic reprogramming to glycolysis (15). Interestingly, the TLR4-mediated glycolytic shift has also assumed considerable attention in the progression of colorectal cancer (CRC) and hepatocellular carcinoma (HRC) (16, 17). The findings strengthen the rationale to investigate the TLR4 signaling pathway as a major upstream trigger of glycolysis in synoviocytes. Further, we also sought to investigate the implications of TIMP1 activation and its contribution to glycolysis, downstream of TLR4 pathway. For long, TIMP1 has been associated with inflammatory outcomes, until the discovery of its cytokine-like functions and its influence in promoting glucose uptake in monocytes (18).

The existing pharmacological options for managing RA include the popular disease-modifying anti-rheumatoid drugs (DMARDs), including non-steroidal anti-inflammatory drugs (NSAIDs), and immunosuppressants, including methotrexate or corticosteroids. Alternative approaches involve the use of biological disease modifiers, including TNF-α or IL-6 inhibitors. However, the prevailing pharmacotherapies used to manage RA suffer from serious adverse effects, including systemic immune suppression, leading to secondary complications and loss of efficacy (19). Thus, there is a consistent need to develop and validate alternative therapies for RA that can potentially minimize the risk of side effects associated with current medications. Over the past decade, our laboratory has validated the pharmacological actions of several natural compounds and polyherbal formulations against RA, strengthening the rationale for their clinical use (20–22).

Habbe Gule Aakh (HGA) is a popular Unani polyherbal formulation composed of extracts from the flowers of *Calotropis procera* (apples of sodom), *Zingiber officinale* (ginger), *Piper nigrum* (black pepper), and *Bambusa arundinaceae* (Indian thorny bamboo). Polyherbal formulations are traditionally used to alleviate joint pain in the management of rheumatoid arthritis to alleviate joint pain. HGA has been previously implicated in alleviating inflammation and possesses analgesic activity in animal models (23). Indeed, the drug has also been investigated in a small cohort of 60 patients to alleviate physical symptoms associated with osteoarthritis (24). However, the clinical premises of HGA remain limited because of insufficient mechanistic validation or scientific rationale. Thus, we investigated the ability of HGA to limit the pathogenic phenotype of LPS-activated SW 982 cells as a first step-up approach to validate the drug against rheumatoid arthritis. Obviously, hindering the TLR4 signaling axis or restoring the altered glycolytic program of synovial cells serves as a solid strategy to combat RA progression.

## 2 Methodology

### 2.1 Reagents and antibodies

HGA was purchased from the National Institute of Unani Medicine (Bangalore, India). The TLR4 inhibitor TAK 242 or Resatorvid (#HY-11109) were purchased from MedChem Express (Princeton, NJ, USA). TLR4 agonist or lipopolysaccharide was purchased from Sigma-Aldrich (St. Louis, MO, USA). The primary antibodies used for immunoblotting and immunofluorescence, HRP- (#AS014), and FITC-conjugated secondary antibodies (#AS011) were purchased from ABclonal (MA, USA). Alexa Fluor 555 (#4413) was purchased from Cell Signaling Technology (Danvers, MA, USA). Glucose Uptake Kit (#G264) and Lactate Assay Kit (#L256) was purchased from Dojindo Laboratories (Kumamoto, Japan). All other chemical reagents used for molecular biology experiments and buffer preparation were procured from HiMedia (Maharashtra, India).

### 2.2 Cell culture and treatment

Human synovial cells or the SW982 cell line were obtained from the National Center for Cell Science, Pune, India. The cells were cultured in DMEM supplemented with 10% Fetal Bovine Serum (FBS) and 1% antibiotic cocktail including 100U/mL penicillin, and 100 ng/mL streptomycin under optimum conditions of 37°C and 5% CO_2._ After attachment, the cells were pre-treated with or without HGA (200μg/mL) for 24h, and TAK242 (1μM) for 1h, followed by stimulation with LPS (2μg/mL) (Sigma-Aldrich, USA) for 24h to activate the TLR4 (Toll like receptor 4).

### 2.3 Cell viability - MTT assay

SW 982 cells were seeded in 96-well plates at a density of 1×10^5^/well for 24h. Following attachment, the cells were treated with HGA at concentrations ranging (20ug/ml to 400ug/ml) for 24h. After incubation, 5 mg/ml MTT was added to each well and the cells were incubated for 4h at 37°C. Following incubation 100μL of DMSO was added to dissolve the formazan crystals, and the color developed after incubation was measured at an absorbance of 570 nm. The percentage of total viable cells per tested drug concentration was compared to the 100% control value.

### 2.4 Cell migration assay

Human synoviocytes or SW 982 cells were seeded at equal density into 6-well plates and cultured to confluency. To study the migration of these synoviocytes, a scratch line was made across the bottom of each well using a 200μL sterile pipette tip, and the treatment was performed as described previously. The cell migration rate was observed at time points 0h, and 24h based on representative images captured at specific time points.

### 2.5 Cell proliferation assay

SW 982 cells were seeded at 50000 per well in a 6-well plate and allowed to adhere overnight at 37°C and 5% CO_2._ The cells were serum-starved in 0.1% FBS-containing medium for 12 h, followed by treatment. Subsequently, SW 982 cells were fixed with 2% paraformaldehyde (PFA), and stained with 0.5% crystal violet for 20 min at room temperature. Images were captured using an EVOS M5000 Fluorescence microscope with a 10X objective. Cell proliferation was quantified relative to that in the untreated group by counting the number of cells in three random fields.

### 2.6 Glucose uptake assay

Glucose uptake in SW 982 cells was assessed using the glucose uptake assay Kit -WST (Dojindo, Japan) following the manufacturer’s protocol. At the end of the treatment, the cell culture supernatants were collected to measure the residual glucose concentration, and the working reagent provided in the kit was added to each well and incubated for 20 min in the dark. The amount of glucose taken up by the cells was quantified by measuring the absorbance at 450 nm, which reflects the formation of WST-based formazan dye. All measurements were performed in triplicate, appropriate blanks and standard controls were included, and values were normalized.

### 2.7 Lactate secretion assay

The secretion of lactate from SW 982 cells was assessed in the culture medium using lactate assay kit (Dojindo, Japan), following the manufacturer’s instructions. Briefly, the standard lactate solutions were prepared by serial dilutions as mentioned. Further, the culture supernatant of treated cells or untreated controls and lactate standards were incubated with working solution provided in the kit. Post 30 minutes of incubation, the absorbance of the respective samples were measured at 450nmm. All measurements were performed in triplicates. The amount of lactate secreted in each sample were interpretated using the calibrated standard curves.

### 2.8 Immunoblotting

SW 982 cells were seeded at equal density and subjected to lysis in RIPA lysis buffer (G Biosciences, USA) supplemented with a cocktail of protease and phosphatase inhibitors following treatment. The lysate was resolved by SDS-PAGE and transferred onto a polyvinylidene fluoride (PVDF) membrane (Immobilon transfer membrane, Merck Millipore, Ireland) by wet transfer. Following 1h blocking in 10% BSA, blots were then washed and probed with antibodies against toll like receptor 4 (#A11226), hexokinase II (#A0994), GLUT1 (#A3330), PKM2 (#A0268), TIMP1 (#A1389), NF-kB p-65 (#A2547) and p-NF-kB p65 (#AP0123), for overnight at 4°C. After overnight incubation with primary antibodies, the membranes were washed twice with TBST and incubated with HRP goat anti-rabbit IgG secondary antibody (#AS014) for 2h at room temperature with gentle shaking. Following this, the blots were washed twice with TBST and once with TBS before being developed using a sensitive chemiluminescence (ECL) solution (Abclonal, USA). The blots were photographed using a ChemiDoc system (Vilber Fx, France). The expression of all the proteins was normalized to that of β-actin.

### 2.9 Real time PCR

The cells were collected, and total RNA was extracted from SW 982 cells using TRIzol (RNAiso Plus, TAKARA), followed by a purity check. 2ug of RNA per sample was used for reverse transcription with the Bio-Rad iScript cDNA synthesis kit (Bio-Rad, Hercules, CA, USA), according to the manufacturer’s protocol. The cDNA was subjected to real-time PCR using the Evagreen Supermix PCR kit (Bio-Rad, Hercules, CA, USA). by using gene-specific primers (**Tables 1**, **2**). The cycling conditions were as follows: initial denaturation at 95°C for 3 min, followed by 39 cycles of 95°C for 10s and 60°C for 30s. A melting curve analysis was performed at the end of each cycle. Gene expression was normalized to that of GAPDH and analyzed using the 2^−△△Ct^ method. The reactions were run in triplicate, and the fold-change values were normalized to GAPDH.

**Table 1:** List of primers used in SW 982 cells.

No.	Gene name	Forward primer	Reverse primer
1	GAPDH	GCTCTCTGCTCCTCCTGTTC	TTCCCGTTCTCAGCCTTGAC
2	TLR4	AAAATCCCCGACAACCTCCC	CACAGCCACCAGCTTCTGTA
3	GLUT1	GTGCCCATGTATGTGGGTGA	CTAGCGCGATGGTCATGAGT
4	PKM2	AGTACTCAGGCTATGGGGCA	CCCTCCCCAGCAGAAAAGAG
5	HK2	GTGAATCGGAGAGGTCCCAC	CAAGCAGATGCGAGGCAATC
6	TIMP1	CATCCTGTTGTTGCTGTGGC	AACTTGGCCCTGATGACGAG
7	MMP-3	TGAGGACACCAGCATGAACC	ACTTCGGGATGCCAGGAAAG
8	RANKL	GAGTTGGCCGCAGACAAGAA	TAGAAGAACAGGGCGACGCT
9	OPG	TAACGTGATGAGCGTACGGG	ACTCCTGCTTGACGTACTGC

**Table 2:** List of primers used in CFA-induced arthritic rats.

No.	Gene name	Forward primer	Reverse primer
1	GAPDH	ACGGGAAACCCATCACCATC	CTCGTGGTTCACACCCATCA
2	TIMP1	TTTCCCTGTTCAGCCATCCC	AATCTGGATTCCGTGGCAGG
3	IL-6	TTCCAGCCAGTTGCCTTCTT	TGGTCTTGGTCCTTAGCCAC
4	TNF-α	GGAGGGAGAACAGCAACTCC	ACTGATGAGAGGGAGCCCAT
5	IL-15	GGCTGGCATCCATGTCTTCA	GCAGTAACTTTGCAACTGGG
6	IL-22	TCCAGCAGCCATACATCGTC	GGCTTTGACTCCTCGGAACA
7	IL-10	GGTAGAAGTGATGCCCCAGG	TTCTTCACCTGCTCCACTGC

### 2.10 Immunofluorescence

The cells were cultured on sterile coverslips, fixed with 2% paraformaldehyde, and permeabilized with 0.1% Triton X-100 in PBS for 15 min. To minimize the nonspecific binding, cells were blocked with 1% goat serum (CST, Danvers, MA, US), followed by the overnight incubation at 4°C with primary antibodies against TLR4 (#A11226), hexokinase II (#A0994), GLUT1 (#A3330), PKM2 (#A0268), TIMP1 (#A1389) and phospho-NF-kB p-65 (#AP0123). After incubation, the cells were washed with PBST and incubated with Alexa Fluor 555- and FITC-conjugated secondary antibodies for 1 h at room temperature. Nuclei were counterstained with Hoechst and coverslips were mounted. The fluorescence images were captured on EVOS M5000 fluorescence microscope. The captured images were quantitatively analyzed for mean fluorescence intensity (MFI) by choosing the region of interest, for biological triplicates using Image J.

### 2.11 Animal ethical approval

All experimental procedures performed in the study were approved by the Institutional Animal Ethical Committee (IAEC) of the Vellore Institute of Technology (VIT), Vellore, India (VIT/IAEC/27/Sep 24/01). Wistar albino rats (150–180 g) were procured and acclimatized in an ambient temperature-controlled environment with a 12h light/dark cycle within the animal house facility of the VIT. The rats were fed an appropriate diet and water. All experimental procedures were performed according to the guidelines prescribed by the Committee for the Purpose of Control and Supervision on Experiments on Animals (CPCSEA), India.

### 2.12 Arthritis induction and drug administration

Arthritis was induced in female Wistar albino rats by administration of 0.1 ml CFA (2 mg/mL) into the right hind paw intradermally on day 0. Following induction, rats were segregated into four groups (n=6 per group). Group I included a subset of healthy non-arthritic rats, which served as the control group. Group II: rats that received CFA induction but no therapeutic intervention, serving as an arthritic control. Group III: To assess the therapeutic potential of HGA, a separate cohort of arthritic rats was orally administered the formulation at a dose of 500 mg/kg body weight from day 11. Group IV: A reference group of arthritic rats was treated intraperitoneally with methotrexate at 1 mg/kg body weight every day from day 11 as a standard drug control.

### 2.13 Assessment of arthritic progression

To monitor arthritis progression, body weight and paw thickness were measured from day 0 and every alternative three days post CFA induction using a digital weighing balance and Vernier caliper. Clinical signs of inflammation and mobility impairment were evaluated daily by visual inspection. Radiographic imaging of the hind limb joint was performed using standardized settings as previously described (21). The severity of arthritis was assessed on a scale of 0-5, based on the extent of joint damage or structural anomalies by two observers in a blinded manner.

### 2.14 Analgesic hot plate test

The analgesic potential of HGA was validated using a hot plate test. Briefly, the experimental rats were positioned in the center of a hot plate, maintained at 55°C, and surrounded by four plexiglass walls. The response time to jump or paw licking was also recorded. Rats were removed from the dish either immediately after licking their hind paws or within 30 s if they did not respond. The experiment was conducted three times at 5-minute intervals, with the medication administered orally 30 min before the procedure.

### 2.15 Histology

Rat joints were collected on 21^st^ day, and preserved in 10% formalin, followed by decalcification process for 2-3weeks in 10% EDTA. After decalcification, tissues were embedded in paraffin and sectioned at 5μm thickness longitudinally. Sections from the joints were stained with hematoxylin and eosin (H&E) to assess tissue morphology, followed by safranin O staining to further evaluate cartilage integrity. Histopathological evaluation was performed in a blinded manner using a standardized semiquantitative scoring system (scale 0-4) to assess key pathological features, such as immune cell infiltration, joint space, pannus formation, and cartilage degradation.

### 2.16 ELISA

The serum concentrations of RANKL and OPG from the experimental rats were quantified using specific ELISA kits, according to the manufacturer’s protocol. Absorbance was measured using an ELISA reader and the concentration was calculated based on a standard curve generated with a known concentration. Briefly, the plates were coated with capture antibodies (1ug/mL), incubated overnight at room temperature. Subsequently, the plates were washed with wash buffer and blocked with 1% BSA. After blocking, the wells were incubated with samples or standards for 2h at room temperature. Serum from the experimental rats was diluted to a ratio of 1:10 and seeded in triplicate. Further, the samples were incubated with detection antibodies (0.25μg/ml) for 2h prior to the addition of avidin HRP conjugate (1:2000) for 30 mins at room temperature. After incubation for 30 mins, the plates were washed thoroughly and developed using ABTS solution (Sigma-Aldrich, St. Louis, USA). Finally, the absorbance values were interpreted using an automated microplate reader at 405 nm with a correction wavelength of 650 nm (Thermo Scientific, USA), and the concentration of cytokines was determined using a standard curve as a reference.

### 2.17 Micro computed tomography assessment

At the end of the treatment, the rats were euthanized, the right hind limbs were excised, and the residual tissue was discarded. Subsequently, intact bone tissue was used to perform computed micro-CT. Briefly, samples were scanned using beam parameters of 40 kV and 100μA, with an isotropic voxel size of 20μm and an integration time of 750ms.

### 2.18 Identification of active ingredients in HGA

The key herbal components of HGA, *Zingiber officinale* Roscoe, *Piper nigrum* Linn, *Calotropis gigantea* Linn, and *Bambusa arundinacea* wild, were selected for analysis. The phytochemical constituents of each ingredient were retrieved from publicly available databases, including Indian Medicinal Plants, Phytochemistry and Therapeutics 2.0 (IMPPAT 2.0), OSADHI, and HERB. Traditional Chinese Medicine Systems Pharmacology Database and Analysis Platform (TCMSP), and PubChem (25–29). Active compounds were screened based on pharmacokinetic parameters, specifically, oral bioavailability (OB) ≥ 30% and drug-likeness (DL) ≥ 0.18. All retrieved phytochemicals were compiled ingredient-wise and duplicate entries were removed using Venny 2.0.

### 2.19 Identification of rheumatoid arthritis-associated targets

Gene expression data related to rheumatoid arthritis were retrieved from the GEO (Gene Expression Omnibus) database using the keyword “rheumatoid arthritis.” Relevant gene chip datasets were merged and normalized using the GEO2R tool. Differentially expressed genes (DEGs) were identified based on the criteria |logFC| > 1 and *p*< 0.05. Additional disease-associated targets were obtained from GeneCards and the Therapeutic Target Database (TTD) (30). All targets from these databases were combined and duplicates were removed to create a comprehensive list of rheumatoid arthritis-related genes. A Venn diagram illustrating the overlap between target sets from each database was generated using Venny 2.0.

### 2.20 Construction of protein–protein interaction networks and core target identification

The interaction network of phytochemicals in HGA was constructed using the STITCH v5.0 database (31), while rheumatoid arthritis-associated gene interactions were mapped via the STRING v10.5 database (32). To ensure high-confidence results, the species was restricted to *Homo sapiens* and interactions were filtered using a stringent confidence threshold of 0.700. Genes identified from differential expression analysis were submitted to STRING, with interaction scores >0.4 considered significant for further analysis. Network visualization and topological analysis were performed using Cytoscape v3.9.1 (33). The CytoHubba plugin was used to identify key hub genes based on six topological parameters: degree centrality (DC), betweenness centrality (BC), closeness centrality (CC), eigenvector centrality (EC), network centrality (NC), and local average connectivity (LAC). Separate subnetworks were generated for the phytochemical targets and RA-related genes to extract the central nodes driving the interaction. Hub genes from both the STITCH and STRING networks were integrated to identify overlapping targets, revealing the key phytochemicals in HGA that potentially interact with critical genes involved in rheumatoid arthritis pathogenesis.

### 2.21 Gene ontology and functional enrichment analysis

To investigate the biological relevance of the identified genes, functional enrichment analysis was performed using Enrichr (34). Gene Ontology (GO) analysis was conducted to categorize the genes into three functional domains: biological processes, cellular components, and molecular functions. Additionally, KEGG pathway enrichment analysis was carried out to identify the key signaling pathways involved in rheumatoid arthritis (35).

### 2.22 Molecular docking

To ensure high-quality input for downstream structural analysis, the 3D coordinates of human CD63 and TIMP1 were obtained from AlphaFold (36), selected based on their high per-residue confidence scores (pLDDT 90.72% for CD63 and 89.6% for TIMP1), while the crystal structure of TLR4 (PDB ID: 2Z63; resolution 2.00 Å) was sourced from the Protein Data Bank (37). Building on our prior network pharmacology analysis, a panel of bioactive phytocompounds: *1,8-Cineole, Borneol, Cadinol, Camphene, Carvacrol, Limonene, Linalool*, and *Thymol* was shortlisted for interaction studies and their (29) standardized 3D conformers were retrieved from PubChem (https://pubchem.ncbi.nlm.nih.gov/) ensuring compatibility with docking algorithms. To localize potential ligand-binding pockets on the protein surface, we employed PrankWeb (https://prankweb.cz/) (38), a tool that leverages deep convolutional neural networks trained on structural features of ligand-binding residues. In parallel, CASTp 3.0 (http://sts.bioe.uic.edu/castp/index.html?4jii) (39) was used to identify topological cavities based on alpha shape theory and solvent-accessible surface mapping. These predicted active sites then guided the docking setup in AutoDock Vina v1.2.0 (https://vina.scripps.edu/) (40), which applies a hybrid scoring function and iterated local search optimization to evaluate binding conformations and affinities. The resulting docked complexes were further analyzed using LigPlot+ and Discovery Studio (https://www.3ds.com/products/biovia/discovery-studio) (41), enabling detailed mapping of key non-covalent interactions, including hydrogen bonds and hydrophobic contacts, which are critical for molecular recognition. To visually assess spatial orientation, residue-level interactions, and conformational fit within the binding pocket, we used UCSF Chimera (https://www.cgl.ucsf.edu/chimera/) and PyMOL (https://www.pymol.org/) (42, 43) for final structural visualization. Together, this integrated in silico workflow provided a rational, structure-driven framework for characterizing the interaction landscape between selected phytochemicals and immune associated proteins, thereby supporting the manuscript’s objective of identifying potential molecular targets for therapeutic intervention.

### 2.23 Phytochemical fingerprinting of Habbe Gule Aakh using LC/HR-MS

Approximately 100mg of the dried extract of HGA was extracted by ultrasonication in 3 mL of ethanol and filtered through 0.22μm syringe filter membrane. The filtrates were collected in vials. The phytoconstituents in the ethanolic fraction were assessed using Waters - Xevo G2- XS - Q ToF High Resolution Mass spectrometer that takes full advantage of the step wave ion optics, XS collision cell, Quant ToF and Ultra-fast electron multiplier and hybrid ADC detector electronics. An ES Ion source in the positive ionization mode was used. Mass parameters were set on the positive mode, Source temperature was 120 ^0^C, Capillary voltage of 1kV, Cone gas flow rate corresponding to 10 Lh^-1^, Desolvation gas flow rate of 800Lh^-1^ and injection volume of 1µL were used. Mass spectra were detected in the range between m/z 100 to1200.Data acquisition and integration were done using the MassLynx4.2 software solution.

### 2.24 Statistical analysis

Data are presented as mean ± standard error of the mean (SEM) derived from three independent studies. GraphPad Prism software (version 8.0.2; GraphPad, MA, USA) Bonferroni multiple comparison post-test and one-way analysis of variance (ANOVA) were used to determine the statistical significance between the experimental groups. P values less than 0.05 were considered as significant.

## 3 Results

### 3.1 HGA blocks TLR4 signaling axis to alter glycolytic program of RA FLS

**Figure 1:**
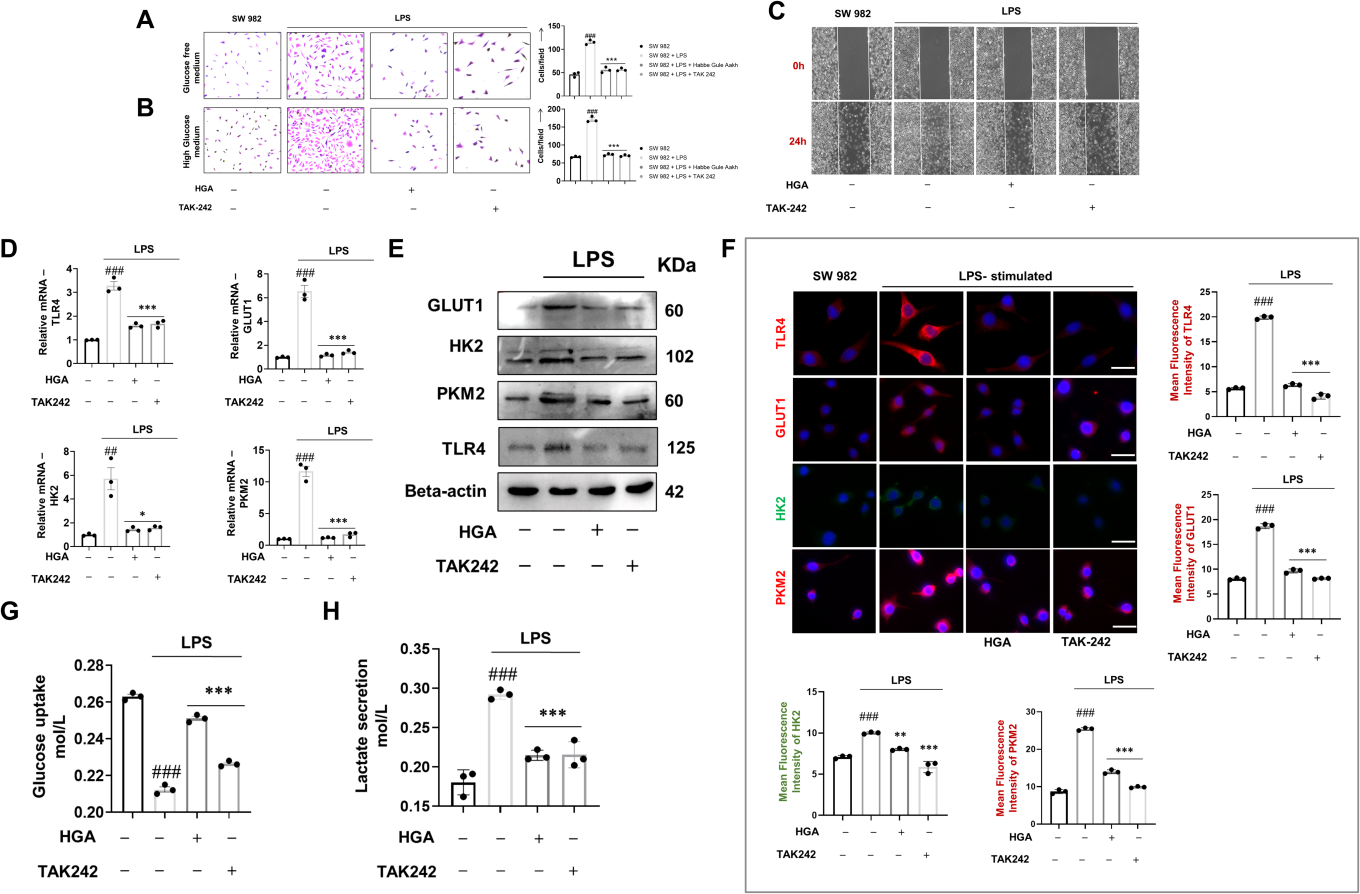
HGA regulates the aberrant glycolytic program of SW 982 cells via TLR4 receptor blockade. To assess the ability of HGA to reverse the glycolytic shift in LPS-activated synoviocytes, a cell proliferation assay was performed by culturing cells under conditions of **(A)** low glucose and **(B)** high glucose. Furthermore, **(C)** the drug formulation was investigated to inhibit the migratory potential of synoviocytes conditioned in a high glucose medium. To ascertain the ability of HGA to regulate glycolysis, we performed **(D)** relative gene and protein levels of TLR4 and key glycolytic pathway proteins including GLUT1, HK2, and PKM2 via **(E)** western blotting and **(F)** immunofluorescence analysis magnification: 40X, Scale Bar: 50μm. Subsequently, we performed **(G)** glucose uptake assay and **(H)** Lactate secretion assay to assess the ability of HGA to inhibit cellular uptake of glucose and thus regulate lactate secretion. The expressed values represent the mean ± SEM of at least three independent experiments. *p < 0.05, **p < 0.01 and ***p < 0.01 verses LPS-stimulated SW 982 cells. *^#^p* < 0.05, *^##^p* < 0.01 and *^###^p* < 0.01 verses SW 982 cells. HGA, Habbe Gule Aakh; LPS, Lipopolysaccharide; TLR4, Toll-like receptor 4; GLUT1, Glucose transporter 1; HK2, Hexokinase 2; PKM2, Pyruvate kinase M2.

As a prerequisite to investigate the *in vitro* efficacy of HGA in altering the tumorigenic character of SW 982 cells in human RA, we assessed its cytotoxicity at increasing concentrations. Our results indicate that the HGA was best tolerated at a dose of 200μg/mL, beyond which dose we observed a significant loss of cell viability (**Supplementary Figure 1**). Furthermore, to assess the ability of HGA to mitigate the hyperproliferative phenotype of LPS-activated SW 982 cells, in the presence or absence of glucose, we performed a cell proliferation assay. As anticipated, HGA regulated proliferation, similar to the TLR-4 antagonist TAK-242 (**Figures 1A, B**). HGA also reversed the invasive phenotype of these cells when cultured in high-glucose medium, mimicking the treatment trend of TAK-242 (**Figure 1C**). We speculated that the tumorigenic characteristics of these synoviocytes are supported by increased glucose turnover or glycolytic flux. To test our hypothesis, we assessed the gene and protein expression of the glycolytic proteins hexokinase 2 (HK2), pyruvate kinase M2 (PKM2), and glucose transporter 1 (GLUT1), downstream of TLR4 signaling. As expected, HGA diminished the expression of TLR4 and glycolytic proteins (**Figure 1D-F**), resulting in diminished glucose uptake (**Figure 1G**). Further, in order to assess the ability of HGA to regulate the glycolysis metabolism, its end-product, lactate was assessed in the culture supernatants. Yet again, HGA consequentially diminished the levels of lactate in the culture supernatant, by regulating glycolytic pathway similar to TAK-242 (**Figure 1H**).

### 3.2 HGA downregulates TIMP1 expression via blockade of TLR4-p65 signaling axis to prevent glucose uptake

**Figure 2:**
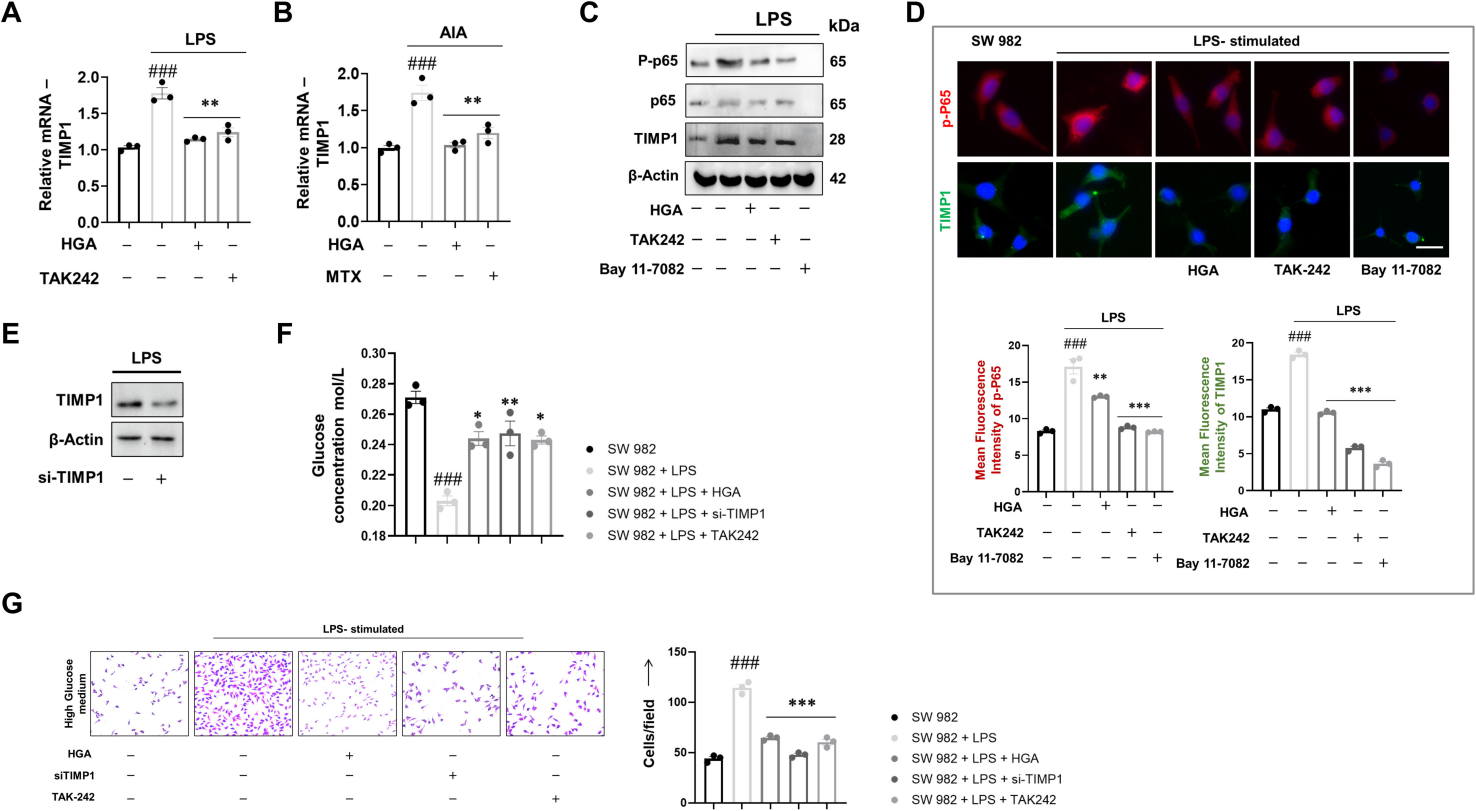
Pharmacological ablation of TIMP1 expression via the TLR4-p65 signaling axis results in diminished glucose uptake. To elucidate the molecular mechanism that drives HGA to control glucose uptake, we assessed the relative expression of TIMP1 in **(A)** LPS-activated synoviocytes and **(B)** animal models of arthritis. To validate the role of the TLR4-p65 signaling axis in promoting TIMP1 expression, we performed relative protein assessment via **(C)** western blotting and **(D)** immunofluorescence using Bay 11-7082, an NF-κB inhibitor, in addition to the existing groups. Magnification: 40X, Scale Bar: 50μm Subsequently, we successfully performed knockdown of TIMP1 via **(E)** si-TIMP1 transfection and assessed the ability of TIMP1 knockdown cells to influence **(F)** glucose uptake and **(G)** cell proliferation, magnification: 20X, Scale bar: 100μm. The expressed values represent the mean ± SEM of at least three independent experiments. *p < 0.05, **p < 0.01 and ***p < 0.01 verses LPS-stimulated SW 982 cells. *^#^p* < 0.05, *^##^p* < 0.01 and *^###^p* < 0.01 verses SW 982 cells. HGA, Habbe Gule Aakh; LPS, Lipopolysaccharide; TLR4, Toll-like receptor 4; TIMP1, Tissue inhibitor of matrix metalloproteinase 1.

Previous reports have shed light on the lesser-known role of TIMP1 in the promotion of glucose uptake. Additionally, reports have also pointed to the increased expression profile of TIMP1 in arthritic joints. Hence, we investigated the gene level expression of TIMP1 in synovial joint samples and synovial cell line downstream of TLR4 signaling. Interestingly, treatment with HGA reversed the gene expression profile of TIMP1 relative to the arthritic model (**Figure 2A**) or LPS-activated SW 982 cells (**Figure 2B**). Simultaneously, our pathway analysis revealed that TIMP1 expression was upregulated downstream of the TLR4-p65 signaling axis. Subsequently, treatment with the p65 inhibitor, Bay 11–7082 alleviated the expression of TIMP1, similar to HGA (**Figures 2C, D**). Furthermore, to assess the effect of TIMP1 in promoting glucose uptake by activated synoviocytes, TIMP1 expression was silenced (**Figure 2E**). Knockdown of TIMP1 via siRNA indicated reduced glucose uptake (**Figure 2F**), resulting in diminished proliferation of SW 982 cells (**Figure 2G**).

Next, to investigate if TIMP1 mediated increase in the glycolytic program could consequentially lead to the expression of osteoclastogenic factors, we assessed the relative gene expression of osteoclastogenic factors MMP9, RANKL, and OPG. As speculated, silencing TIMP1 diminished the expression of MMP9 and RANKL (**Supplementary Figures 2A, B**), while improving the transcriptional program for OPG (**Supplementary Figure 2C**), which acts as a soluble decoy for RANKL, thereby preventing cartilage degradation or osteoclastogenesis.

### 3.3 HGA alleviates physical features of arthritis in an experimental model

**Figure 3:**
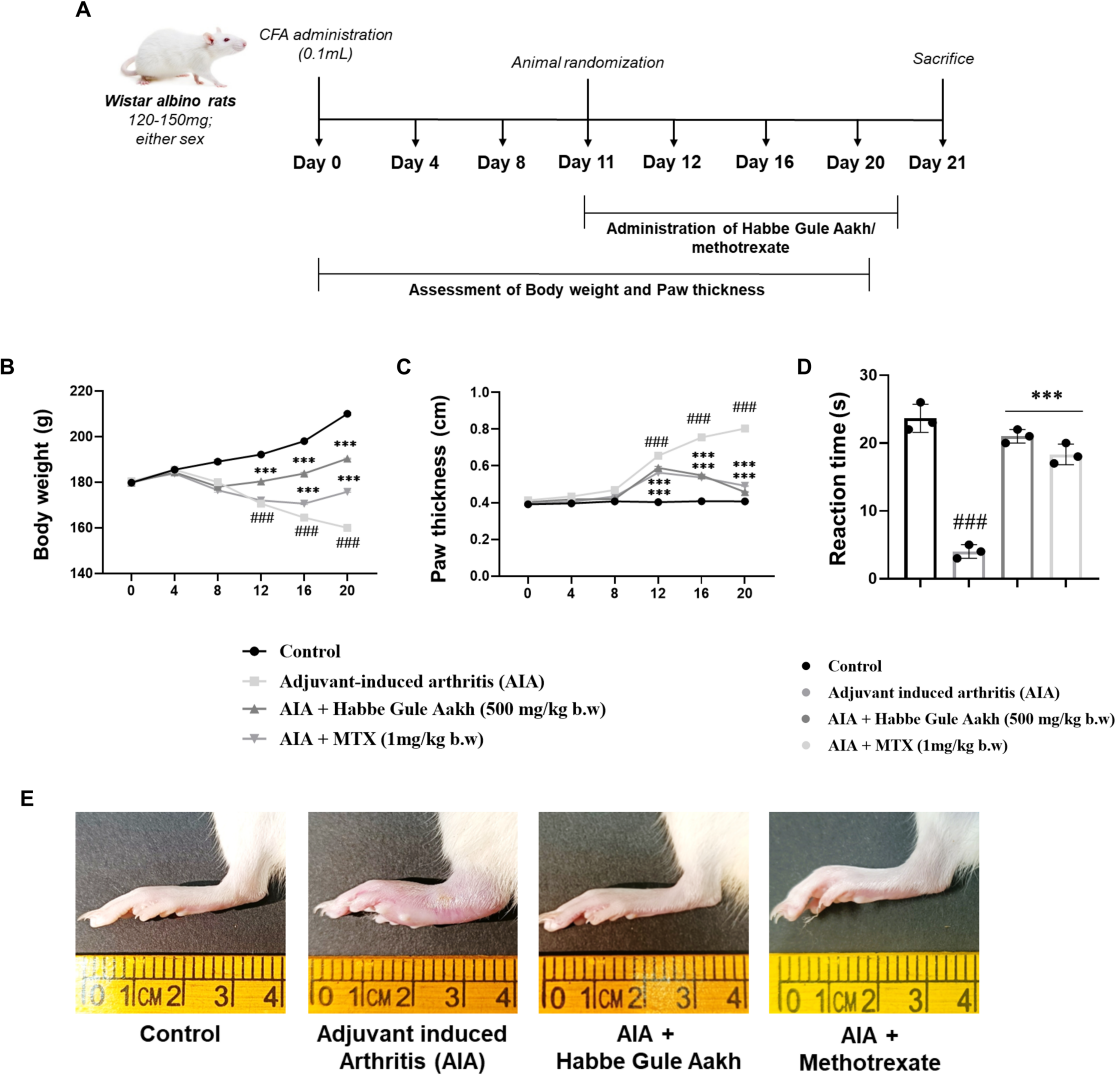
HGA alleviates the physical features of disease progression in CFA-induced arthritic rats. We adopted a preclinical approach to gain better insights into the anti-arthritic potential of HGA. **(A)** The CFA-induced arthritis experimental timeline lapsing over 21 days was used for our *in vivo* investigations. Subsequently, assessment of physical parameters, including **(B)** body weight and **(C)** paw volume, was recorded on the indicated days. **(D)** An analgesic hot-plate test was performed to evaluate the analgesic properties of HGA in arthritic rats. At the end of treatment, **(E)** paw images were captured, indicating paw edema or inflammation. The expressed values represent the mean SEM of at least three independent experiments. *p < 0.05, **p < 0.01 and ***p < 0.01 verses non-treated arthritic control. *^#^p* < 0.05, *^##^p* < 0.01 and *^###^p* < 0.01 verses healthy control. HGA, Habbe Gule Aakh; MTX, methotrexate; CFA, Complete Freund's Adjuvant; AIA, Adjuvant-induced arthritis.

Furthermore, to ascertain the anti-arthritic efficacy of HGA, we established a CFA-induced arthritis model to assess and compare the efficacy of the polyherbal formulation with that of the standard drug of choice (**Figure 3A**). Body weight assessments and paw volume measurements on alternate days were indicative of disease induction or resolution. HGA effectively sustained the improved body weight and reduced paw volume (**Figures 3B, C**). Additionally, the analgesic properties of HGA were evaluated in experimental rats using the hot-plate test. Interestingly, administration of HGA also improved pain tolerance in arthritic rats, demonstrating efficacy on par with the standard drug methotrexate (**Figure 3D**). Subsequently, paw images were captured at the end of induction and treatment. Treatment with HGA revealed significant resolution of paw edema, similar to methotrexate (**Figure 3E**).

### 3.4 HGA prevents cartilage degradation and protects joint architecture

**Figure 4:**
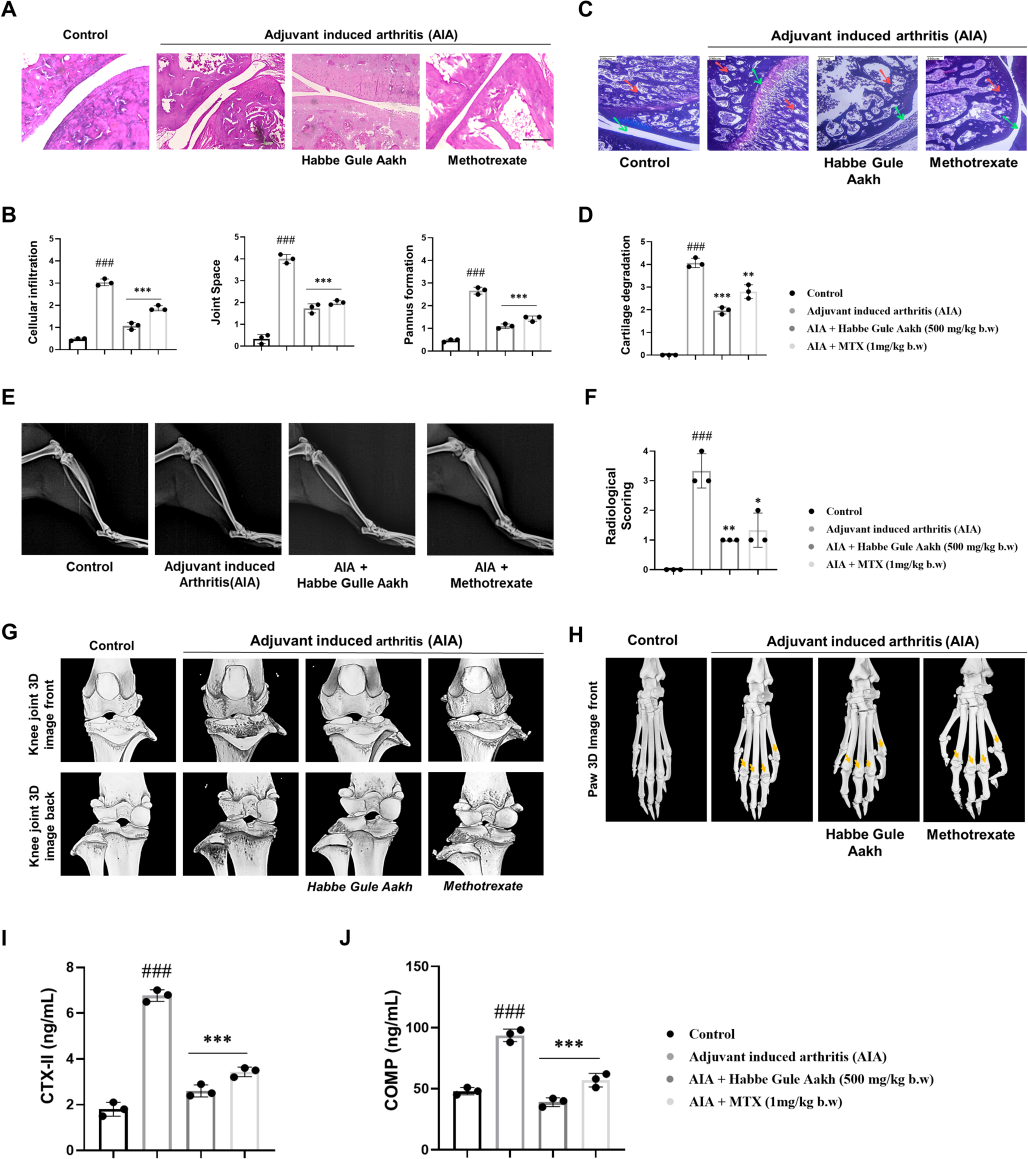
HGA prevents cartilage degradation and preserves joint architecture in CFA-induced arthritic rats. **(A)** Histological staining of synovial joints was performed on experimental rats to qualitatively assess **(B)** cellular infiltration, pannus formation, and joint space on a scale of 0-5. **(C)** Safranin O staining was performed on the synovial joints of experimental rats to assess **(D)** cartilage degradation. **(E, F)** Radiographic assessments and micro-computed tomography were performed, indicating cartilage degradation and bone damage in the **(G)** knee and **(H)** paw joints; the yellow arrow indicates regions of bone erosion in the diarthrodial joints of the paw. To quantitatively assess the extent of cartilage degradation, the serum levels of **(I)** CTX-II and **(J)** COMP were assessed by ELISA. The expressed values represent the mean ± SEM of at least three independent experiments. *p < 0.05, **p < 0.01 and ***p < 0.01 verses non-treated arthritic control. *^#^p* < 0.05, *^##^p* < 0.01 and *^###^p* < 0.01 verses healthy control. HGA, Habbe Gule Aakh; MTX, methotrexate; CFA, Complete Freund’s Adjuvant; AIA, Adjuvant-induced arthritis; CTX-II, C-Terminal Cross-linked Telopeptides of Type II Collagen; COMP, Cartilage Oligomeric Matrix Protein.

Histological assessment of the synovial joint indicated reduced cellular infiltration, pannus formation, and improved joint space after HGA administration (**Figures 4A, B**). Safranin staining also demonstrated diminished cartilage degradation upon treatment with HGA (**Figures 4C, D**). Subsequently, radiological imaging of the knee joints revealed diminished bone deformity in HGA treated arthritic rats, demonstrating significant efficacy parallel to the administration of methotrexate (**Figures 4E, F**). To evaluate the changes in the synovial cartilage or knee joint, we performed computed micro-tomography, which offers greater resolution of joint damage. We observed significantly improved resolution of joint damage in HGA-treated arthritic rats compared with in those arthritic rats. Interestingly, although methotrexate provided potent regulation of key pathogenic mediators in RA, HGA fared better in the current model (**Figures 4G, H**).

Further, to quantitatively measure or assess the extent of cartilage degradation, we measured the levels of C-terminal telopeptide of type II collagen (CTX-II) and cartilage oligomeric matrix protein (COMP), in the serum of experimental rats. CTX-II, is a popular prognostic marker at the early stages of RA, indicating loss of bone mineral density and cartilage breakdown. COMP forms a part of the cartilage extracellular matrix, and its elevated levels further reaffirm cartilage degradation (44, 45). As observed in the analysis of knee joints in computed micro-tomography, the levels of CTX-II and COMP were found to be elevated in arthritic mice, while treatment with HGA and MTX reversed this trend, potentially altering the cartilage degradation mechanism, thereby preserving structural integrity of the knee joints (**Figures 4I, J**).

### 3.5 HGA resolves joint damage by regulating the cytokine milieu

**Figure 5:**
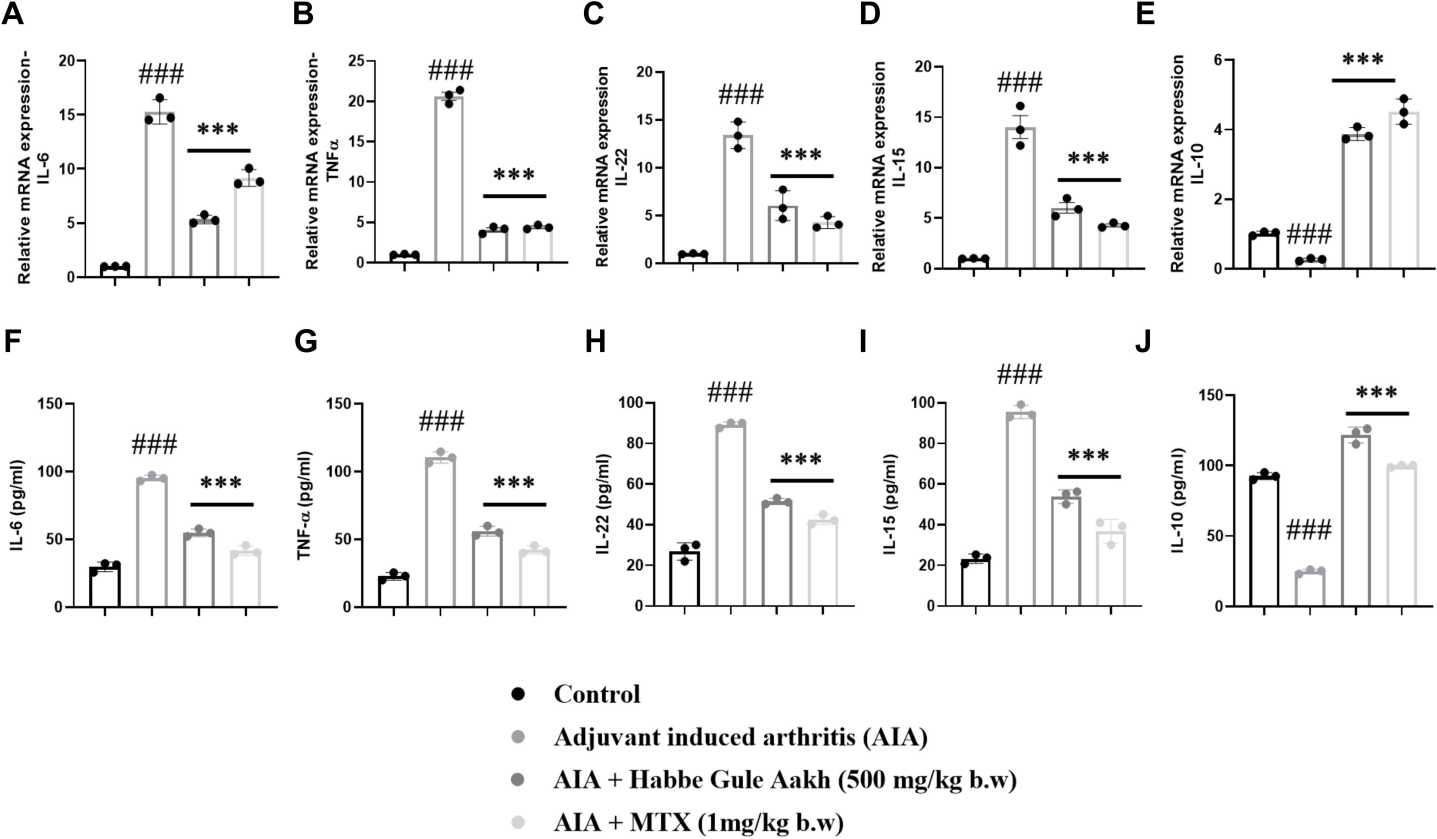
HGA ablates disease severity by regulating the inflammatory milieu in arthritic rats. The relative gene levels of pro-inflammatory cytokines **(A)** IL-6, **(B)** TNF-α, **(C)** IL-15, **(D)** IL-22, and anti-inflammatory cytokine **(E)** IL-10 in joint synovial tissue were assessed based on RT-PCR. Simultaneously, the serum supernatants of experimental rats were collected to measure the levels of these cytokines via **(F–J)** ELISA. The expressed values represent the mean ± SEM of at least three independent experiments. *p < 0.05, **p < 0.01 and ***p < 0.01 verses non-treated arthritic control. *^#^p* < 0.05, *^##^p* < 0.01 and *^###^p* < 0.01 verses healthy control. HGA, Habbe Gule Aakh; MTX, methotrexate; IL, interleukin.

Relative gene expression analysis of synovial tissue showed that HGA reduced the expression profile of key pro-inflammatory cytokines in the RA synovium, including IL-6, TNF-α, IL-22, and IL-15, while markedly improving the transcriptional program of the key regulatory cytokine IL-10 (**Figures 5A–E**). Simultaneously, our analysis of the key cytokines in the secretome or serum samples also revealed similar trends (**Figures 5F-J**).

### 3.6 Computational analysis reveals strong association between HGA and glycolytic targets in rheumatoid arthritis

**Figure 6:**
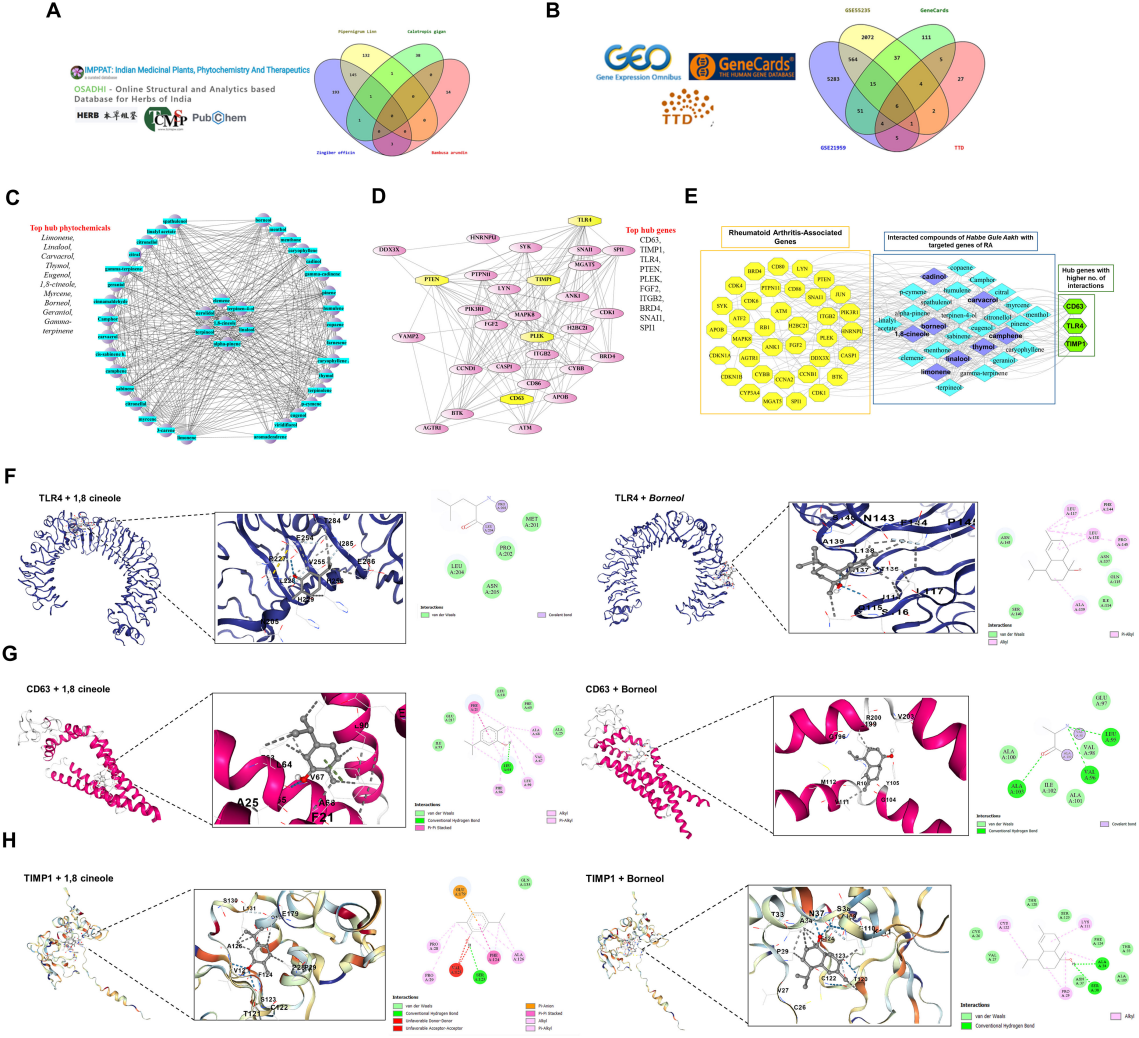
Phytochemical and gene interaction network analysis of HGA ingredients in rheumatoid arthritis. To support our *in vitro* findings, we adopted a network pharmacology-based approach to validate HGA activity against key glycolytic targets in RA. **(A, B)** Data collection and Venn diagrams showing overlapping phytochemical and disease-associated genes. **(C, D)** Cytoscape network visualization of phytochemical and gene interactions, highlighting the top 10 phytochemicals and hub genes. **(E)** Overall integrated network analysis of *Habbe gule Aakh* phytochemicals and Rheumatoid associated genes revealed key interactions with CD63, TLR4, and TIMP1. Note: Yellow nodes represent targeted genes, sky blue nodes represent phytochemicals, and green nodes indicate hub genes with a higher number of interactions with phytochemicals purple in color. Molecular docking results showing the interactions of two main active phytocompounds 1,8-Cineole and Borneol with key rheumatoid arthritis (RA)-associated target proteins: TIMP1, CD63, and TLR4. **(F)** the binding of both compounds with TIMP1, **(G)** shows interactions with CD63, and **(H)** with TLR4. The docking models highlight the binding conformations and key interface residues involved in each protein-ligand complex, supporting the potential therapeutic relevance of these compounds in RA. TLR4, Toll like receptor 4, TIMP1, Tissue Inhibitor of matrix metalloproteinase 1, CD63, Cluster of differentiation 63.

A total of 4,582 active ingredients were identified from the IMPPAT, OSADHI, HERB, TCMSP, and PubChem databases using the screening criteria of oral bioavailability (OB) ≥ 30% and drug-likeness (DL) ≥ 0.18. The distribution of compounds was as follows: 531 from *Zingiber officinale* Roscoe, 510 from *Piper nigrum* Linn, 90 from *Calotropis gigantea* Linn, and 25 from *Bambusa arundinacea* Willd. After removing duplicates and ingredients without the corresponding gene targets, 220 unique active compounds were retained for further analysis (**Figure 6A**). Gene expression profiles related to rheumatoid arthritis were retrieved from the GEO database, specifically the GSE21959 and GSE55235 datasets. GSE21959 includes synovial tissue samples from patients with RA and healthy controls, while GSE55235 contains peripheral blood mononuclear cells (PBMCs) from patients with RA and healthy individuals. Differential gene expression analysis was performed using GEO2R, with thresholds set at |logFC| > 1 and *p*< 0.05. A total of 7,300 differentially expressed genes were identified: 2,072 from GSE21959 and 5,283 from GSE55235. To complement this, 120 RA-related genes were obtained from the GeneCards database and 50 from the Therapeutic Target Database (TTD). After merging and removing duplicates, 1,800 unique RA-associated target genes were identified (**Figure 6B**).

The active compounds from HGA were submitted to the STITCH database to identify compound-target interactions. The resulting network consists of 757 nodes and 1,987 edges. This network was visualized using Cytoscape and key topological parameters were analyzed using the CytoHubba plugin. Based on the degree centrality, the top 10 active compounds were identified: *Limonene, Linalool, Carvacrol, Thymol, Eugenol, 1,8-Cineole, Myrcene, Borneol, Geraniol, and Gamma-Terpinene*. These compounds showed the highest number of interactions and were considered to be the most influential in the therapeutic potential of *Habbe Gule Aakh* (**Figure 6C**). For rheumatoid arthritis genes, a protein–protein interaction (PPI) network was constructed using the STRING database, resulting in 800 nodes and 1,534 edges. The network was visualized using Cytoscape, and hub genes were identified using CytoHubba. The top ten hub genes included CD63, TIMP1, TLR4, PTEN, PLEK, FGF2, ITGB2, BRD4, SNAI1, and SPI1, each showing high connectivity and potential relevance in RA pathogenesis (**Figure 6D**).

To elucidate the most influential interactions, a combined network integrating the top-ranked compounds and hub genes was constructed. The analysis revealed robust associations between key RA-related genes CD63, TLR4, and TIMP1 and highly interactive compounds, such as *Cadinol, Carvacrol, Borneol, Thymol, Camphene, Limonene, Linalool, and 1,8-Cineole*. These compounds exhibited the highest degree values and centrality scores, indicating significant targeting potential. The strong connectivity within this integrated network highlights these compound–gene pairs as critical modulators of the therapeutic mechanism of HGA against rheumatoid arthritis (**Figure 6E**).

Further downstream, the identified constituents were docked against TLR4, TIMP1 and CD63, to assess relatively stable binding affinities against target proteins. The structural validation of TLR4, CD63, and TIMP1 was carried out using resolution metrics and per-residue model confidence scores. Conserved functional residues were mapped and selected for docking. For TLR4, the binding site comprised residues 117, 136, 137, 138, 140, 167, 202, 204, and 205. For CD63, conserved residues included 14, 18, 21, 24, 25, 28, 64, 67, 68, 86, 90, 93, 97, 101, 102, 213, 216, 217, and 220. In TIMP1, residues 33, 36, 37, 147, 150, 151, and 152 formed the predicted active region. To evaluate phytochemical interactions, 24 docking simulations were performed with eight selected ligands against the three proteins. Docking centers were defined as *(24.4323, -39.3759, 4.0756)* for TLR4, *(-13.5649, -2.9795, 10.1844)* for CD63, and *(5.7750, 7.5244, -5.4987)* for TIMP1. The respective conservation scores for these binding pockets were 0.42 (TLR4), 11.0 (CD63), and 2.07 (TIMP1). Among the phytochemicals, 1,8-Cineole and Borneol demonstrated the most favorable binding affinities across all three proteins as mentioned in (**Table 3**), making them prime candidates for further analysis.

**Table 3:** Molecular properties and binding affinities of selected phytochemical ligands against target proteins CD63, TLR4, and TIMP1.

Ligands	Molecular formula	Molecular weight (g/mol)	Energy minimization	Binding affinity of a protein (kcal/mol)		
			(EM)	CD63	TLR4	TIMP1
*1,8-Cineole*	C_10_H_18_O	154.25	78.47	-6.3	-5.2	-5.4
*Borneol*	C_10_H_18_O	154.25	224.46	-6.5	-5.5	-6.0
*Cadinol*	C_15_H_26_O	222.37	466.88	-4.4	-4.8	-5.0
*Camphene*	C_10_H_16_	136.23	102.50	-5.0	-4.5	-5.0
*Carvacrol*	C_10_H_14_O	150.22	344.57	-4.6	-4.5	-5.3
*Limonene*	C_10_H_16_	136.23	354.78	-4.8	-4.4	-4.6
*Linalool*	C_10_H_18_O	154.25	95.97	-5.4	-5	-5.3
*Thymol*	C_10_H_14_O	150.22	118.54	-4.6	-4.3	-4.5

In the TLR4–1,8-Cineole complex, residues P227, L228, H239, V255, P256, E284–E286 form a hydrophobic cavity, with additional van der Waals and covalent-like contacts involving MET201, PRO202, LEU204, and ASN205. TLR4–Borneol binds within a groove of LEU117 to PHE144 through π-alkyl, alkyl, and van der Waals interactions, overlapping the conserved region. In CD63–1,8-Cineole, the ligand is stabilized by PHE21, ALA25, PHE65, LEU64, VAL67, ALA68, and LEU90, along with conserved contacts with LEU18, GLU217, and ILE93. CD63–Borneol interacts through hydrogen bonds and van der Waals forces with R109, Y105, G104, M112, and surrounding hydrophobic residues like VAL96, ALA100, and ILE102. For TIMP1–1,8-Cineole, key residues C122–A126 form hydrogen bonds, π–π stacking, and van der Waals interactions, with additional contacts to GLU179 and F124, despite some unfavorable donor-donor or acceptor-acceptor overlaps. TIMP1–Borneol is stabilized by hydrogen bonding with SER123, ASN37, and ALA34, and hydrophobic interactions involving PHE124, CYS122, and PRO29. Altogether, these findings validate the precise and conserved nature of the ligand-binding sites in TLR4, CD63, and TIMP1. The strong and consistent binding of 1,8-Cineole and Borneol across all three proteins supports their potential as multi-target therapeutic candidates, particularly for modulating immune and inflammatory pathways (**Figures 6F–H**).

Subsequently, preliminary phytochemical assessments were carried out using LC-HRMS to identify the phytoconstituents present in the ethanolic fraction of HGA (**Supplementary Table 1**). Further, LC-HRMS analysis identified distinct compound signatures at different retention times using positive mode (**Supplementary Figure 4**). However, we observed that rigorous phytochemical analysis would be required using extracts with different solvents to fully recapitulate and understand the phytoconstituents of Habbe Gule Aakh.

### 3.7 Network pharmacology of HGA demonstrated molecular basis for altered glucose metabolism

**Figure 7:**
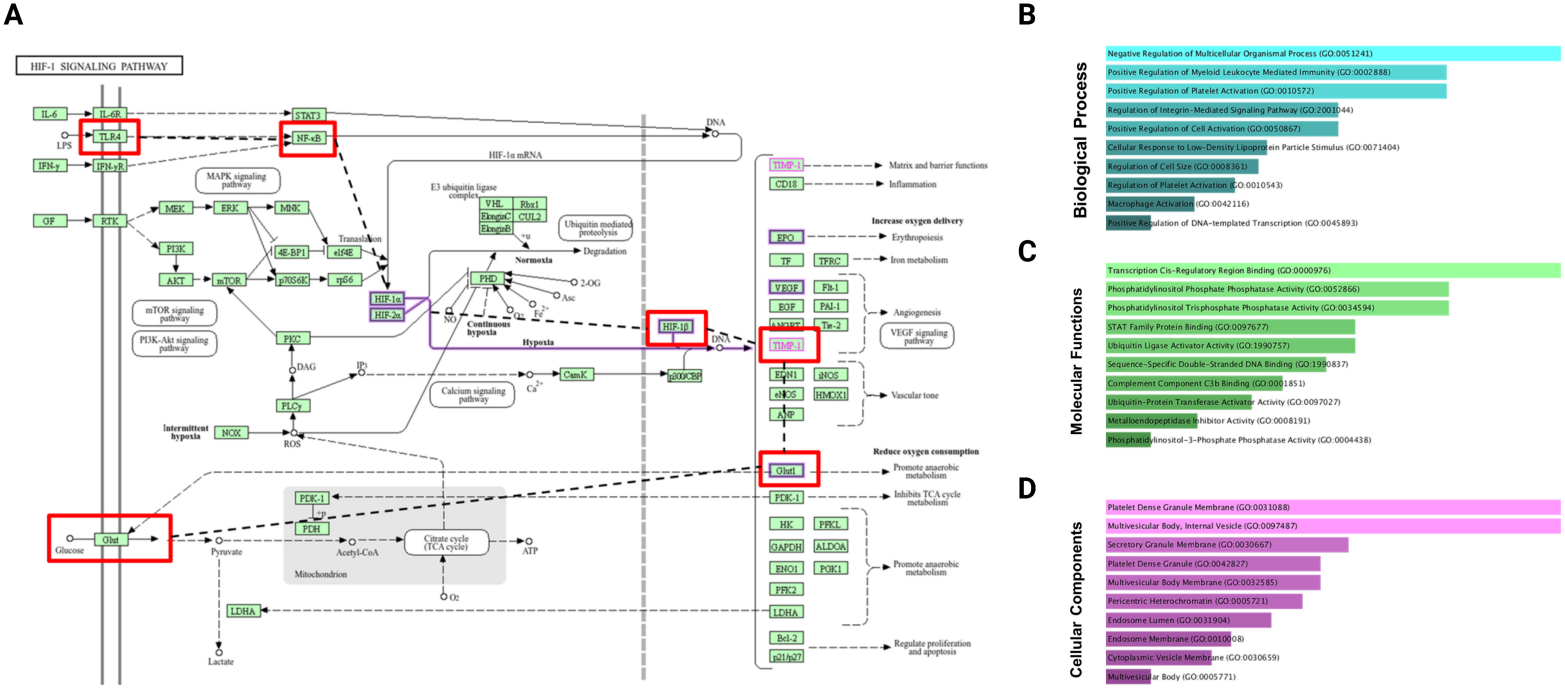
KEGG pathway analysis and Gene Ontology (GO) enrichment of TIMP1 at elevated glucose levels. **(A)** KEGG pathways, **(B)** biological processes, **(C)** molecular functions, and **(D)** cellular components.

In the HIF-1α signaling pathway, TLR4 activation leads to NF-κB signaling, which promotes the expression of hypoxia-responsive genes, such as Glut1, enhancing glucose uptake to support inflammatory cell metabolism. Simultaneously, TIMP1, regulated by hypoxic and inflammatory cues, contributes to extracellular matrix remodeling and inflammation. Gene ontology analysis supports this interplay: biological processes such as I-κB phosphorylation (p = 0.0005999) link TLR4 to NF-κB activation, whereas TIMP1 is involved in the negative regulation of multicellular organismal processes (p = 0.0001470) and trophoblast cell migration (p = 0.0004999). Cellular component terms associated TIMP1 with platelet alpha granules (p = 0.006589) and molecular functions confirmed its role as a metalloendopeptidase inhibitor (p = 0.001300), as shown in (**Figures 7A–D**), highlighting its significance in the pathogenesis of RA. Further, to correlate the findings of KEGG pathway analysis, substantiating HIF1α activation to stabilize TIMP1 expression and glycolytic pathway enzymes, we assessed the transcriptional status of HIF1α utilizing HGA, TLR4 pathway inhibitor and Bay11-1082. Expectedly, treatment with HGA ablated the activation of HIF1α. Notedly, TLR4 pathway blockade with TAK-242 and inhibition of TLR4-p65 signaling axis using Bay11–7082 revealed relatively significant outcomes in regulating the expression of HIF1α (**Supplementary Figure 3A**).

## 4 Discussion

Pharmacological ablation of TLR4 signaling is shown to alleviate pathological outcomes in animal models of rheumatoid arthritis (46). Based on preclinical studies, TLR4 antagonists have been developed as a strategy to combat RA disease severity. However, despite the promising outcomes of TLR4 antagonists, pharmacological targeting of the innate immune receptor has been obscured by its limited efficacy in clinical trials (47). Recent reports from our laboratory have revealed promising therapeutic implications of TLR4 signaling blockade in experimental models of rheumatoid arthritis using TAK-242. TAK-242 reversed the hyperplastic phenotype of fibroblast-like synoviocytes, subverting cartilage degradation in a CFA-induced animal model of RA (10). Thus, in the current study, we decoded the therapeutic targeting of TLR4 signaling using HGA, a polyherbal unani formulation, with TAK-242 as our positive control. HGA offers distinctive advantages as a drug formulation that is traditionally used in RA standard care. The polyherbal formulation empowers promising clinical outcomes for pain management (23, 24). The tumorigenic phenotype of activated fibroblast-like synoviocytes is a key pathological feature of RA. Previous studies have attributed enhanced glycolysis or glucose uptake to the aggressive behavior of fibroblast-like synoviocytes (FLS), culminating in synovitis (48, 49).

To assess the efficacy of the drug in modulating the tumorigenic characteristics of synovial cells, we conducted a series of *in vitro* experiments utilizing SW 982 cells. These cells are frequently used in the study of synovitis in rheumatoid arthritis (RA) (50). Our current reports decode how HGA orchestrates the pharmacological reversal of the glycolytic program mediated by the TLR4 signaling pathway. HGA reversed the expression profile of the rate-limiting glycolytic enzymes, hexokinase 2 (HK2) and pyruvate kinase M2 (PKM2), thereby disrupting the energy metabolism of human synovial cells. Alternatively, HGA diminished the expression of glucose transporter 1 (GLUT1), thereby impacting glucose uptake. Skewed glucose metabolism also improves lactate production, which is symbolic of the glycolytic shift. The metabolic alterations affected by HGA reinforced the non-invasive phenotype of synovial cells, curtailing cell proliferation and migration. The pharmacological implications of HGA are similar to those of the inhibition of the TLR4 signaling pathway by TAK-242. Understandably, ablation of glycolytic program has previously shown to alleviate arthritic progression, For instance, 2-deoxyglycose (2-DG), an inhibitor of the rate limiting glycolytic pathway enzyme hexokinase II, has earlier being demonstrated to block the advancement of adjuvant-induced arthritis. Parallelly, glycolytic pathway regulation by 2-DG has also shown to alter macrophage polarization via AMPK signaling pathway (51). On an even intriguing note, 2-DG could modulate TLR4 pathway activation to redefine macrophage polarization, and inflammatory microenvironment (52). The cumulative findings thus bridged the gap between TLR4 pathway activation and glycolytic shift, almost persuading a symbiotic or closely inter-related crosstalk. This further led us to speculate that the HGA may modulate the TLR4 signaling pathway to regulate the aberrant glycolytic program. As expected, HGA altered the expression profile of the TLR4 receptor.

Consistent with our reports that suggest an upward trend in glycolysis to support the energy demand of activated synoviocytes, we were curious to understand the mechanism that could influence such an outcome. Transcriptomic reports have suggested enhanced expression of tissue inhibitors of matrix metalloproteinase 1 (TIMP1) in inflamed RA synovium (53). TIMP1 is a matrix metalloproteinase protein that intrinsically inhibits MMP9 activity by its N-terminal domain (54). However, emerging studies have pointed out much wider implications for TIMP1, demystifying its nomenclature (18). TIMP1 has been shown to promote glucose uptake in monocytes via its C-terminal domain. Furthermore, the C-terminal domain of TIMP1 has been shown to promote proinflammatory cytokine profiles in monocytes (55). Interestingly, another report indicated that TIMP knockout animal models showed reduced energy gains in contrast to TIMP1 wild-type mice fed a high-fat diet (56). These findings led us to speculate that abundant TIMP1 in the RA synovium might contribute to altered glycolytic metabolism in human synoviocytes. As expected, our initial findings showed improved TIMP1 in LPS-activated SW 982 cells. This finding implies that LPS stimulation can lead to TIMP1 production. Alternatively, the treatment of activated cells with HGA diminished TIMP1 levels. Importantly, the expression profile of TIMP1 was reduced after treatment with TAK242, reinforcing the direct role of TLR4 signaling in promoting TIMP1 expression.

KEGG pathway analysis revealed that the NF-κB pathway downstream of TLR4 led to TIMP1 production under hypoxic conditions. NF-κB also serves as a popular transcription factor in RA, attracting therapeutic strategies targeting its expression (57). The NF-κB signaling pathway also reinforces critical events in RA disease progression, including inflammation, tissue destruction, and synovial hyperplasia (44). This led us to speculate that inhibition of the p65 pathway downstream of TLR4 signaling could restore TIMP1 expression. As expected, activated synoviocytes demonstrated decreased TIMP1 expression upon p65 inhibition. This indicates that the TLR4-p65 signaling pathway drives TIMP1 expression in activated or hyperproliferative human synovial cells. Supposedly, silencing TIMP1 also diminished glucose uptake and consequently led to diminished proliferation of these cells. These findings suggest a more pronounced role of TIMP1 in the energy metabolism of human RA synoviocytes. Our findings suggest a renewed dimension of TIMP1; however, further studies are required to shed more light on the proactive role of TIMP1 in the progression of human RA. RNA sequencing of TIMP1 knockout synovial cells or TIMP1 knockin/knockout animal models can further yield insights into its role in modulating energy metabolism.

The tumorigenic phenotype of activated synoviocytes also leads to excessive production of matrix metalloproteinases and other soluble pathogenic mediators including receptor activator of nuclear factor kappa B ligand (RANKL). The sustained pro-inflammatory milieu can exaggerate cartilage degradation and bone erosion. Knockdown of TIMP1 or treatment with HGA similarly regulated the expression of MMP9 and RANKL while improving the level of osteoprotegerin or OPG, which acts as a soluble decoy to RANKL. Consistent with our findings, we investigated the disease-modulating effect of the HGA in animal models of arthritis. As expected, HGA mitigated the pathological effects of CFA-induced arthritis. Our histopathological assessments revealed reduced cellular infiltration and pannus formation upon treatment with HGA. Safranin O staining confirmed reduced cartilage degradation, along with radiographic and micro-computed tomography assessments. Importantly, treatment with HGA also regulates the expression profile of key inflammatory mediators in RA. Notedly, in an attempt to further elucidate and underscore the anti-arthritic effects of Habbe Gule Aakh, we utilized network pharmacology approach to pin down the interactome of key phytoconstituents with RA target genes. Interestingly, our integrated network analysis revealed a higher interaction of HGA phytoconstituents with TLR4, TIMP1, and CD63, verifying our *in vitro* observations utilizing the RA human synoviocytes cell line. It is important to note that CD63 serves as a cell-surface binding partner for TIMP1, facilitating downstream signal transduction (58). Previously, the key phytoconstituents of HGA have been obtained from entries corresponding to individual plant constituents in a polyherbal formulation using publicly available databases. Furthermore, upon KEGG pathway analysis it was observed that HIF1α activated and stabilized TIMP1 expression downstream of TLR4-p65 signaling axis. Hence, we decoded this causal relationship to pin significant mechanistic cues. Accordingly, we observed that activation of TLR4 or p65 signaling pathway to reinforce HIF1α expression. However, further research is warranted for any prospective relationship between the RA hypoxic microenvironment and TIMP1 activation. Investigating the immunomodulatory effect in animal models of arthritis may reveal the therapeutic effects of the HGA. Moreover, clinical investigations utilizing polyherbal formulations can reinforce the potency of HGA in the RA settings.

## 5 Conclusion

Overall, our multi-pronged approach utilizing *in vitro*, *in vivo*, and in silico analyses reaffirms the therapeutic potential of HGA as a pharmacological agent for the management of rheumatoid arthritis. Thus, the outcomes of the current study revitalize the clinical implications of HGA, strengthening the basis for its wider integration in RA care.

## Data Availability

The original contributions presented in the study are included in the article/**Supplementary Material**. Further inquiries can be directed to the corresponding author.

## Supplementary material

Supplementary Figure 1 Initially, the cytotoxicity of Habbe Gule Aakh (HGA) was assessed in LPS-activated RA human synoviocytes or SW 982 cell line by performing **(A)** MTT assay.

Supplementary Figure 2 To validate the ability of TIMP1 to influence cartilage degradation, we assessed its ability to alter the relative gene and protein expression of osteoclastogenic factors RANKL and OPG upon gene knockdown by performing **(A–C)** RT-PCR of MMP3, RANKL, and OPG. The expressed values represent the mean ± SEM of at least three independent experiments. **p* < 0.05, ***p* < 0.01 and ****p* < 0.01 verses LPS-stimulated SW 982 cells. *^#^p* < 0.05, *^##^p* < 0.01 and *^###^p* < 0.01 verses SW 982 cells. HGA, Habbe Gule Aakh; MTX, methotrexate; AIA, Adjuvant-induced arthritis; MMP3, Matrix metalloproteinase 3; RANKL, Receptor Activator of Nuclear factor Kappa B Ligand; OPG, Osteoprotegerin.

Supplementary Figure 3 The relative gene level expression of hypoxia-inducible factor 1α was assessed using **(A)** RT-PCR. The expressed values represent the mean ± SEM of at least three independent experiments. **p* < 0.05, ***p* < 0.01 and ****p* < 0.01 verses LPS-stimulated SW 982 cells. *^#^p* < 0.05, *^##^p* < 0.01 and *^###^p* < 0.01 verses SW 982 cells.

Supplementary Figure 4
**(A)** The liquid chromatogram showing different retention times for the ethanolic fraction of HGA. **(B–I)** The mass spectrum corresponding to retention times 2.143, 2.788, 2.941, 3.093, 3.262, 3.451, 3.637 and 4.520.
