# Supplementary material for: Habbe Gule Aakh prevents glycolytic program and alleviates disease progression in a rheumatoid arthritis animal model
Source: Front Immunol. 2025 Aug 29;16:1633061. doi: 10.3389/fimmu.2025.1633061 (PMC12425913; doi:10.3389/fimmu.2025.1633061)
Supplement: Supplementary file 1 [file Supplementaryfile1.docx]

**Supplementary Table.1: Predicted Compounds of Habbe Gule Aakh based on LC/HRMS analysis**

| **Sl. No** | **RT** | **Theoretical m/z** | **Observed m/z** | **Predicted compound** | **Ontology** |
| --- | --- | --- | --- | --- | --- |
| 1. | 2.143 | 405.2272 | 405.2159 | Calotropagenin | Pregnane glycoside |
| 2. | 2.143 | 449.1078 | 449.0998 | Isoorientin | Flavonoid |
| 3. | 2.788 | 274.1438 | 274.2586 | Piperlonguminine | Alkaloid |
| 4. | 2.788 | 588.2626 | 588.2729 | Uscharin | Cardenolides |
| 5. | 2.941 | 286.1438 | 286.1324 | Piperine | Alkaloid |
| 6. | 2.941 | 288.1594 | 288.1430 | Piperanine | Alkaloid |
| 7. | 3.093 | 312.1594 | 312.1499 | Piperettine | Alkaloid |
| 8. | 3.093 | 314.1387 | 314.1635 | N-feruloyltyramine | Phenolic |
| 9. | 3.262 | 224.2009 | 224.0927 | Pellitorine | Amide alkaloid |
| 10. | 3.262 | 224.2009 | 224.1856 | Neo pellitorine B | - |
| 11. | 3.262 | 225.1849 | 225.1886 | Linalool isobutyrate | monoterpenoid |
| 12. | 3.262 | 340.1907 | 340.1844 | Dehydropipernonaline | Piperidine alkaloid |
| 13. | 3.262 | 362.3417 | 362.1684 | (2E,4E,14Z)-N-Isobutyl-2,4,14-eicosatrienamide | Amide alkaloid |
| 14. | 3.451 | 344.2220 | 344.2158 | Piperolein B | Piperidine alkaloid |
| 15. | 3.451 | 358.2377 | 358.2321 | Piperchabamide D | - |
| 16. | 3.451 | 597.1450 | 597.3149 | Apigenin 6-C-glucoside 8-C-arabinoside | Flavonoid |
| 17. | 3.637 | 370.2377 | 370.2463 | Piperchabamide B | Amide |
| 18. | 3.637 | 384.2533 | 384.2675 | Guineensine | Amide |
| 19. | 4.520 | 139.0309 | 139.9639 | Hydroxybenzoic acid | Phenolic acid |
| 20. | 4.520 | 334.3104 | 334.2951 | Piperine | Alkaloid |
| 21. | 4.520 | 336.3216 | 336.3016 | (2E,4E)-N-isobutyl-2,4-octadecadienamide | Amide alkaloid |
| 22. | 4.520 | 346.3104 | 346.2945 | Piperlonguminine | Amide alkaloid |
