# Supplementary figures and images for: Habbe Gule Aakh prevents glycolytic program and alleviates disease progression in a rheumatoid arthritis animal model
Source: Front Immunol. 2025 Aug 29;16:1633061. doi: 10.3389/fimmu.2025.1633061 (PMC12425913; doi:10.3389/fimmu.2025.1633061)

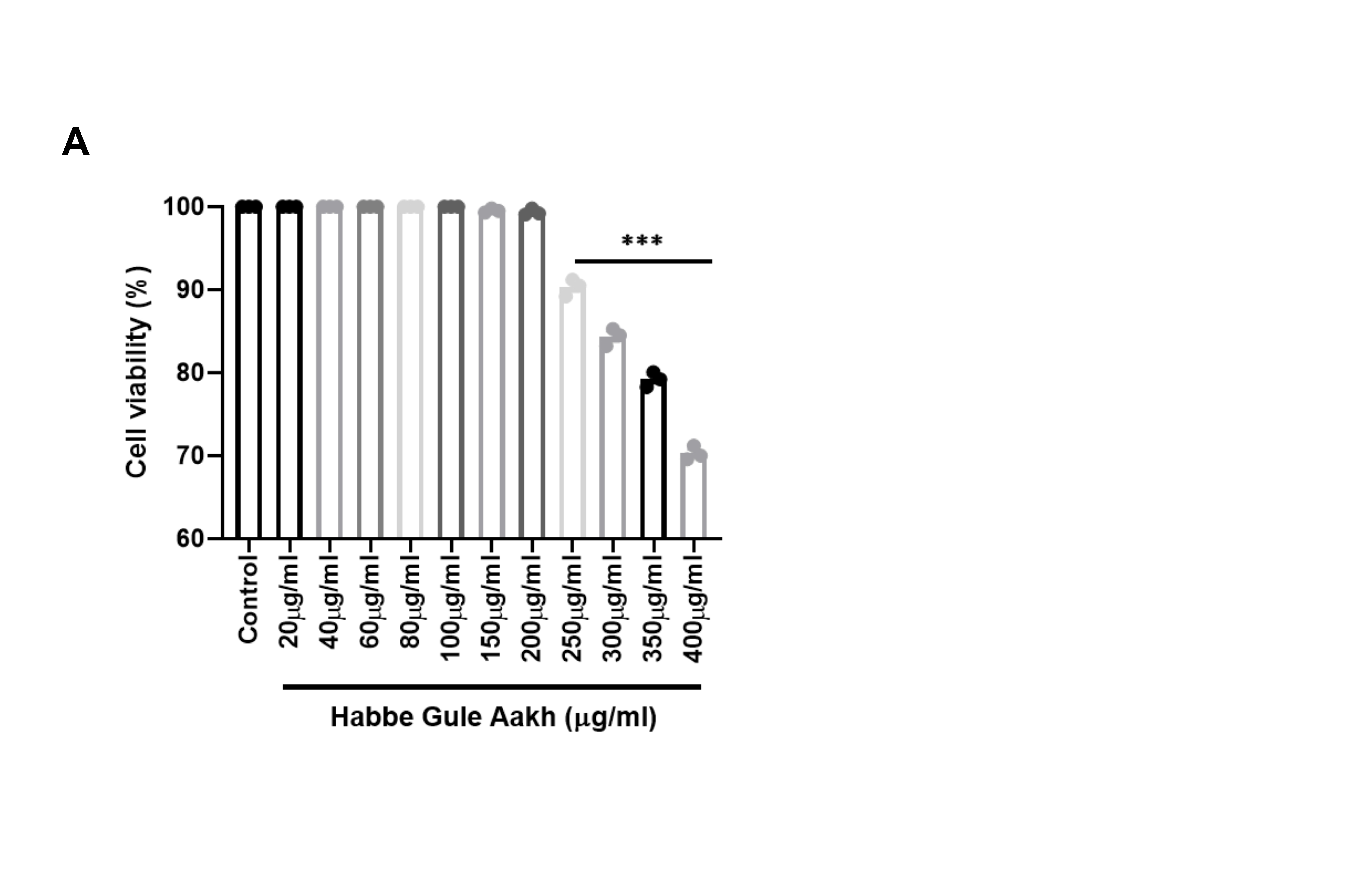

Supplement: Supplementary Figure 1 — Initially, the cytotoxicity of Habbe Gule Aakh (HGA) was assessed in LPS-activated RA human synoviocytes or SW 982 cell line by performing (A) MTT assay. [file Image1.tiff]

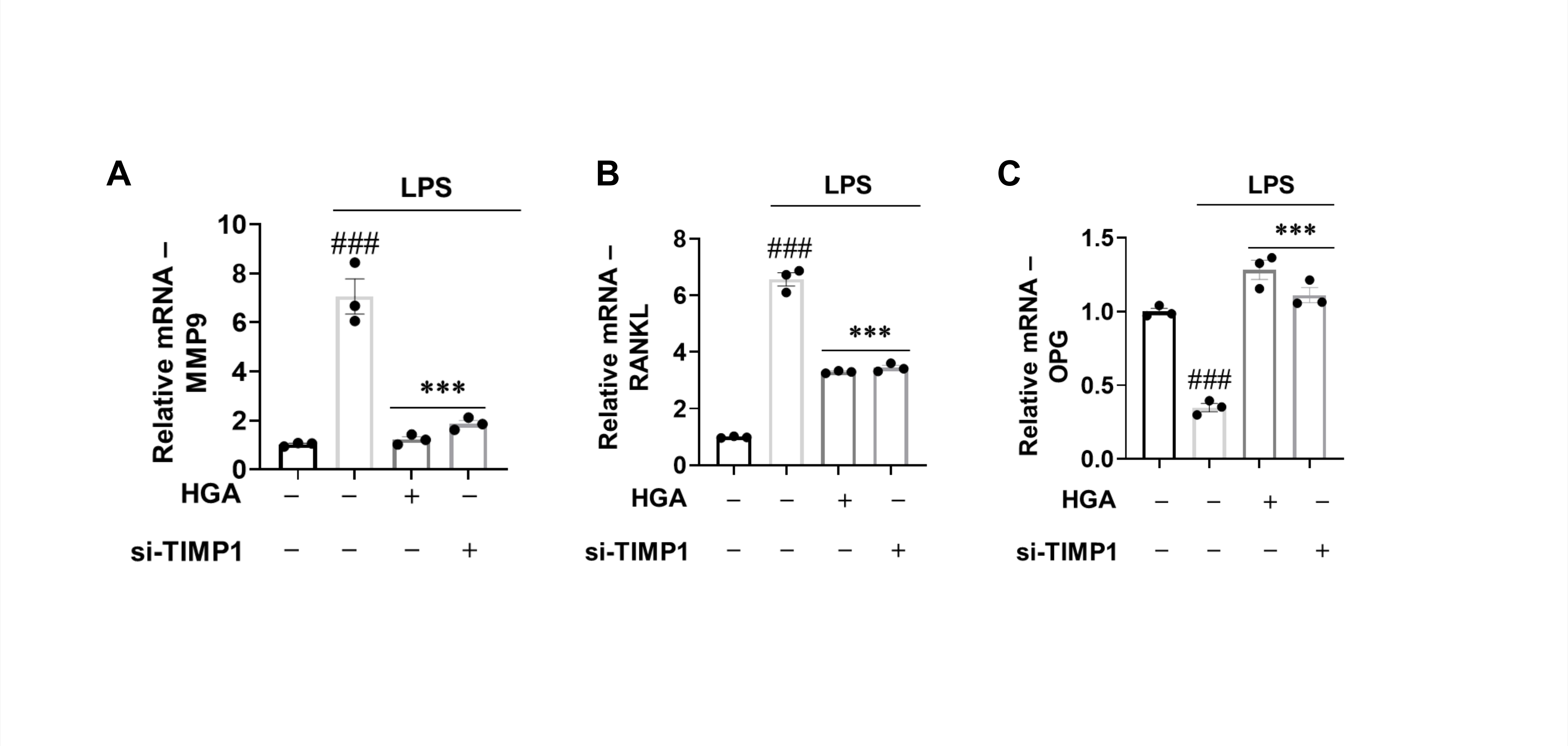

Supplement: Supplementary Figure 2 — To validate the ability of TIMP1 to influence cartilage degradation, we assessed its ability to alter the relative gene and protein expression of osteoclastogenic factors RANKL and OPG upon gene knockdown by performing (A–C) RT-PCR of MMP3, RANKL, and OPG. The expressed values represent the mean ± SEM of at least three independent experiments. *p < 0.05, **p < 0.01 and ***p < 0.01 verses LPS-stimulated SW 982 cells. #p < 0.05, ##p < 0.01 and ###p < 0.01 verses SW 982 cells. HGA, Habbe Gule Aakh; MTX, methotrexate; AIA, Adjuvant-induced arthritis; MMP3, Matrix metalloproteinase 3; RANKL, Receptor Activator of Nuclear factor Kappa B Ligand; OPG, Osteoprotegerin. [file Image2.tiff]

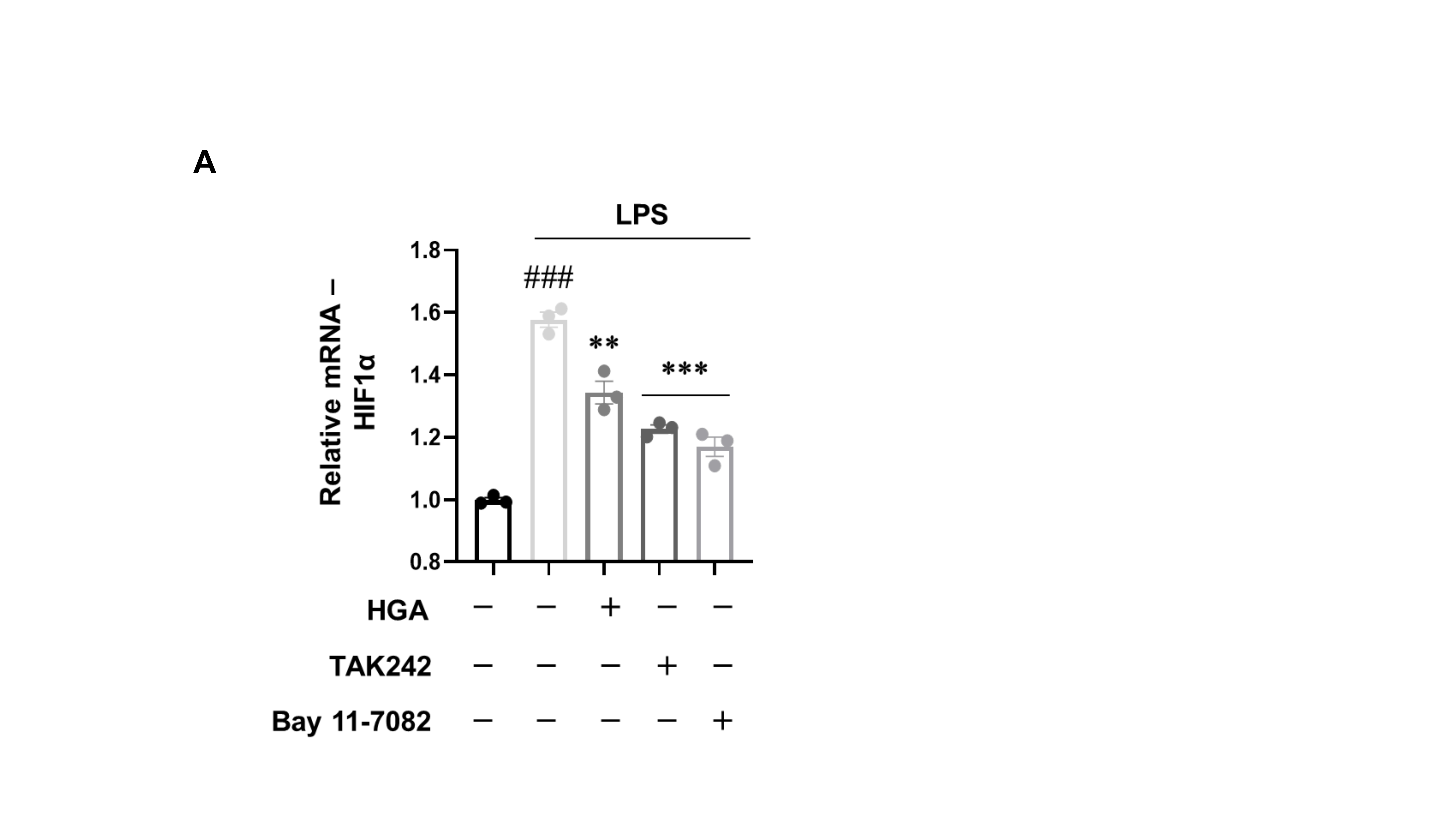

Supplement: Supplementary Figure 3 — The relative gene level expression of hypoxia-inducible factor 1α was assessed using (A) RT-PCR. The expressed values represent the mean ± SEM of at least three independent experiments. *p < 0.05, **p < 0.01 and ***p < 0.01 verses LPS-stimulated SW 982 cells. #p < 0.05, ##p < 0.01 and ###p < 0.01 verses SW 982 cells. [file Image3.tiff]

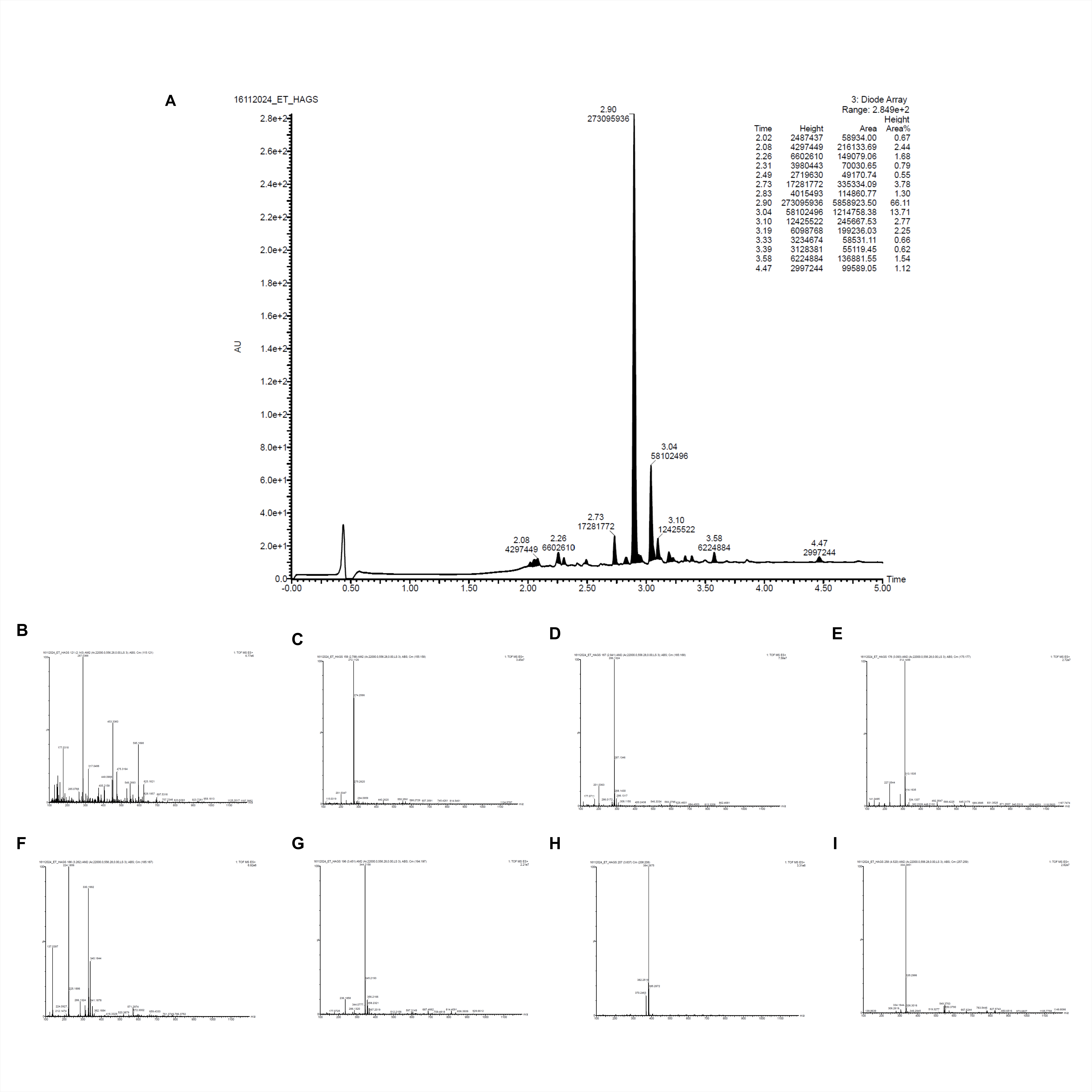

Supplement: Supplementary Figure 4 — (A) The liquid chromatogram showing different retention times for the ethanolic fraction of HGA. (B–I) The mass spectrum corresponding to retention times 2.143, 2.788, 2.941, 3.093, 3.262, 3.451, 3.637 and 4.520. [file Image4.tiff]
